# Predicting the Stepwise Growth of Lithium Clusters Around Sulfur Species (S and S_2_ ) From the Topology of the Electron Localization Function

**DOI:** 10.1002/jcc.70503

**Published:** 2026-09-09

**Authors:** Mohammad Esmaïl Alikhani

**Affiliations:** ^1^ Sorbonne Université, CNRS, De la Molécule Aux Nano‐Objets: Réactivité, Interactions et Spectroscopies, MONARIS Paris France

**Keywords:** DFT, ELF, five‐coordinate, hyperlithiated sulfur, inner and outer valence domains, localized and delocalized valence electrons

## Abstract

In this work, we present a theoretical investigation of sulfur‐doped lithium clusters, namely Li_*n*_S and Li_*n*_S_2_ (*n* = 1–10), with the aim of elucidating the mechanism governing the stepwise growth of lithium around sulfur centers. The equilibrium geometries of all clusters were first determined using density functional theory (DFT), and their electronic structures were subsequently analyzed through the topology of the electron localization function (ELF). The ELF analysis revealed the presence of two distinct types of topological valence domains: an inner domain fully localized on the sulfur atom and an outer domain delocalized over the lithium framework. We show that the growth of the lithium cluster is governed by the subtle interplay between these two domains. Most importantly, the combined topology of the inner and outer valence domains provides a predictive framework that not only identifies the preferred insertion site for each incoming lithium atom but also explains the progressive emergence of successive coordination shells, thereby leading systematically to the most stable structures throughout the growth process. The present findings open the way to a predictive description of lithium growth around larger sulfur species.

## Graphical abstract

Hyperlithiated sulfur species: an ELF analysis.

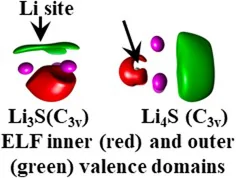

## 1 Introduction

Lithium, in combination with sulfur and oxygen, plays a central role in modern electrochemical energy‐storage technologies [1, 2, 3, 4, 5]. In idealized Li–S batteries, elemental sulfur at the cathode is reduced successively to a series of lithium polysulfides (Li_2_S_*n*_, 2 ≤ *n* ≤ 8), ultimately yielding Li_2_S_2_ and Li_2_S as the final discharge products.

The discharge mechanism involves a complex sequence of sulfur‐chain reduction, fragmentation, and lithiation reactions, giving rise to a rich network of intermediate species. Consequently, considerable theoretical and experimental efforts have been devoted to elucidating the structural, electronic, and thermodynamic properties of isolated lithium polysulfides, together with their interactions with solvents, electrolytes, and electrode materials [6, 7, 8, 9, 10, 11].

By contrast, significantly less attention has been paid to the direct interactions between lithium polysulfides and nanoscale lithium particles, represented at the molecular level by lithium clusters. These clusters constitute valuable model systems for probing the fundamental chemistry occurring at lithium‐metal interfaces and during the early stages of lithium deposition [12]. Despite their relevance to Li–S electrochemistry, both theoretical and experimental investigations of the reactions between lithium polysulfides and finite lithium clusters remain remarkably scarce. In particular, the interactions of representative polysulfides, including Li_2*n*_S_*n*_ (*n* = 1–8), with lithium clusters have received only limited attention [10, 13, 14, 15]. A systematic investigation of these systems is expected to provide fundamental insight into the evolution of chemical bonding, electron transfer, and structural reorganization accompanying the progressive reduction of sulfur species under lithium‐rich conditions. Such knowledge is essential for establishing a molecular‐level understanding of the elementary processes governing the behavior of lithium–sulfur systems.

Lithium, despite being a monovalent element, is capable of forming not only predominantly ionic compounds but also more unconventional bonding motifs, including hydrogen‐like bonds [16, 17, 18, 19, 20, 21], and multicenter bonds reminiscent of those encountered in boron chemistry [22, 23, 24, 25, 26, 27].

The unusual bonding schemes exhibited by lithium‐rich compounds have already been highlighted in a number of hyperlithiated species, suggesting that lithium can stabilize excess electrons in a way that deviates considerably from conventional ionic bonding. In the early 1990s, two hyperlithiated sulfur molecules, Li_3_S and Li_4_S, were detected experimentally in mass spectrometry [28]. The measured dissociation energies demonstrate that both electron‐rich species are thermodynamically stable with respect to lithium atoms loss. To rationalize these observations, the authors perform ab initio molecular orbital calculations showing that Li_3_S possesses C_3v_ symmetry, while Li_4_S adopts a C_2v_ cage‐like structure. The calculated electronic configurations reveal that the highest occupied molecular orbitals are largely delocalized over the lithium framework rather than being localized on the sulfur atom. This delocalization is a key factor in stabilizing the excess valence electrons.

The present work aims to address this gap through a comprehensive quantum‐chemical investigation of the interactions between stoichiometric lithium polysulfides, Li_2__*n*_S_*n*_ (*n* = 1–2), and finite lithium clusters, Li_*m*_ (*m* ≤ 8). While the structures and energetics of the resulting complexes are characterized in detail, the primary objective is to uncover the electronic principles governing the sequential growth of lithium around sulfur centers. Particular attention is devoted to the evolution of the Li–S bonding environment, the redistribution of electronic density, and the reorganization of interatomic interactions as the lithium framework expands. A central aspect of this study is to assess whether the topology of the electron localization function (ELF) can not only rationalize the observed structures but also predict the preferred sites for the incorporation of additional lithium atoms. By establishing a direct relationship between ELF topology and cluster growth, this work provides a unified molecular‐level interpretation of sulfur reduction under lithium‐rich conditions.

## 2 Computational Details

All first‐principles calculations were performed using the Gaussian 09 quantum‐chemical packages [29]. Stationary points on the potential energy surface were optimized within the framework of density functional theory using the widely employed B3LYP‐GD3(BJ) exchange–correlation functional [30, 31, 32] including the D3 version of Grimme's dispersion with Becke–Johnson damping [33]. The Pople triple‐ζ quality basis Set 6‐311+G(2d), extended with polarization and diffuse functions, was employed for all atoms [34, 35].

To validate the geometric and electronic results obtained at the B3LYP‐GD3(BJ) level, two highly symmetric isomers of Li_4_S, possessing C_2v_ and C_3v_ symmetry, respectively, were further optimized using the CCSD(T)/6‐311+G(2d) method.

Chemical bonding pattern was analyzed within the framework of the electron localization function (ELF) [36]. The partitioning of the molecular space in terms of non‐overlapping space‐filling domains was carried out using the TopMod package [37]. Visualization of ELF isosurfaces was performed using UCSF Chimera [38].

## 3 Results and Discussion

### 3.1 Li_*n*_S (*n* = 1–10): Structural Growth and Bonding Pattern

It has long been recognized that lithium oxide (Li_2_O) is a linear molecule, whereas its heavier congener, lithium sulfide (Li_2_S), adopts a bent equilibrium geometry. This fundamental structural difference has commonly been ascribed to the higher polarizability and lower electronegativity of sulfur compared with oxygen, leading to significant differences in the electronic structure and bonding characteristics of the two molecules [39, 40, 41]. An analogous structural trend is found for the corresponding trilithium species: while Li_3_O exhibits a planar D_3_h geometry, Li_3_S adopts a pyramidal C_3v_ structure. These observations suggest that replacing oxygen by sulfur induces profound changes in the nature of the Li–*X* (*X* = O and S) interaction, raising fundamental questions regarding the balance between ionic and covalent contributions to the chemical bond [28, 42].

**FIGURE 1:**
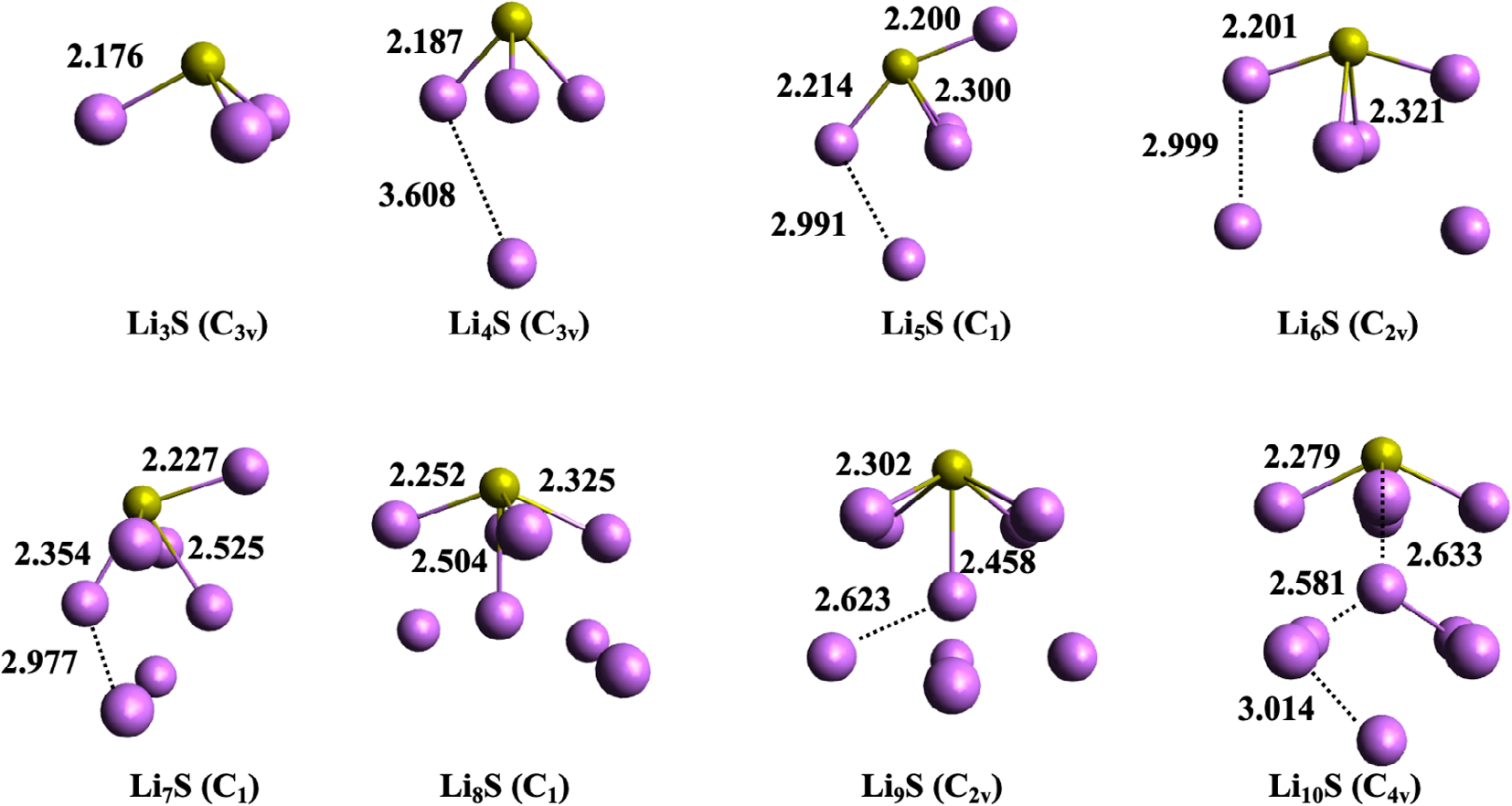
Schematic representation of the optimized geometries of Li_*n*_S (*n* = 3–10), together with the most relevant bond lengths. The purple and yellow spheres represent lithium and sulfur atoms, respectively.

The fully optimized geometric structures are depicted in Figure 1. Corresponding cartesian coordinates are reported in the Supporting Information (SM‐1).

Upon the successive addition of lithium atoms to the initial Li_2_S molecule—which obeys the octet rule—the interaction energy varies with cluster size, ranging from 24.1 kcal/mol for Li_7_S to 36.4 kcal/mol for Li_3_S.

A close examination of the structures shown in Figure 1 reveals that, for *n* = 10, coordination extends up to the third shell. Interestingly, the formation of these coordination shells as *n* increases from 3 to 10 does not follow a regular pattern. For example, the second shell appears already in Li_4_S, whereas the addition of a fifth lithium atom completes the first coordination shell. As will be shown below, this apparently irregular shell growth is in fact governed by the way valence electrons are accommodated within the cluster, allowing the sulfur atom to preserve its octet throughout the growth process.

**FIGURE 2:**
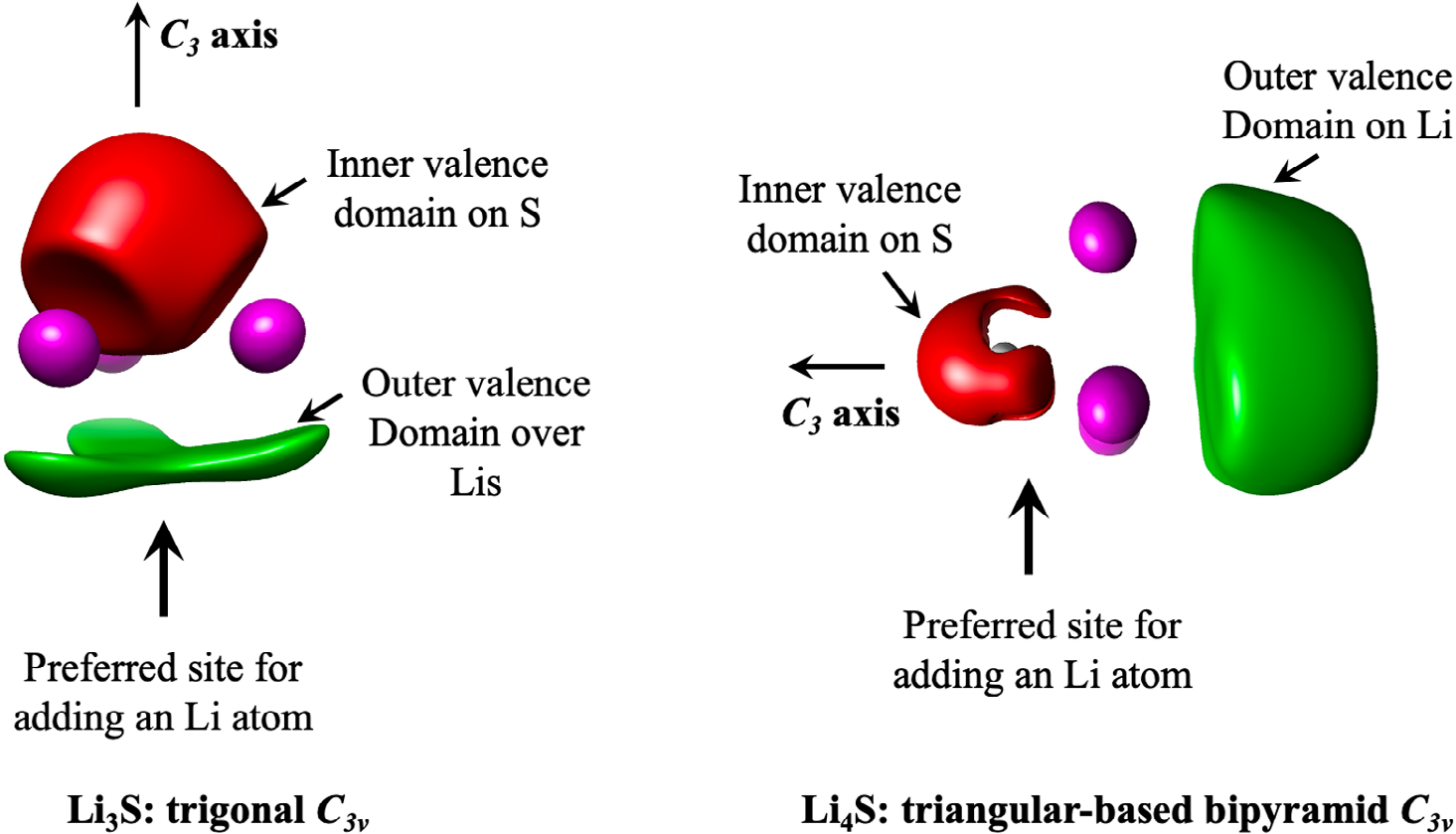
Inner and outer ELF valence domains for trigonal Li_3_S (*η*(*r*) = 0.60) (left) and triangular‐based bipyramid Li_4_S (*η*(*r*) = 0.85) (right). Lithium atoms are represented by purple spheres. The sulfur atom is surrounded by the inner valence domain (red).

In the case of Li3*X* (*X* = O, S), the central atom is formally surrounded by three lithium atoms and the system might therefore be regarded, at first sight, as hypervalent. Such an interpretation, however, is not justified [43, 44, 45, 46]. As previously emphasized for several sulfur‐ and fluorine‐containing compounds, the population of the central atom remains slightly below the octet, amounting to approximately 7.9 electrons for both oxygen and sulfur. The additional electron is not localized on the central atom but is instead delocalized over the three peripheral lithium atoms. The electron localization function (ELF) topology of Li_3_S provides compelling support for this interpretation. Figure 2 (left panel) clearly shows that eight of the nine valence electrons of Li_3_S are localized on the sulfur atom (red color), whereas the ninth electron is predominantly delocalized over the three lithium atoms (green color), away from the sulfur center. Owing to their spatial arrangement, these two electron‐rich regions give rise to two distinct topological domains, referred to as the inner and outer domains. Throughout this work, the terms “inner topological valence domain” and “outer topological valence domain” refer to the ELF basins associated with the sulfur center and the peripheral valence electron density, respectively [47, 48].

The outer valence domain constitutes the preferred site for accommodating a fourth lithium atom, naturally leading to a Li_4_S structure with C_3v_ symmetry. This prediction is inconsistent with the C_2v_ geometry proposed by Kudo et al. [28]. This discrepancy arises because Kudo et al. considered only the C_2v_ structure. Geometry optimizations of both isomers using two independent computational approaches, B3LYP‐D3(BJ)/6‐311+G(2d) and CCSD(T)/6‐311+G(2d), clearly demonstrate that the C_2v_ isomer corresponds to a local minimum on the potential energy surface. It is connected to the global minimum (C_3v_) through a transition state associated with a very low activation barrier of only 0.5 kcal mol^−1^ at the B3LYP‐D3(BJ)/6‐311+G(2d) level.

In the C_3v_ structure of Li_4_S, the sulfur atom is three‐coordinate, while the fourth lithium atom belongs to the second coordination shell. As a consequence of this structural arrangement, eight of the 10 valence electrons are localized within the inner topological domain (red), centered on the sulfur atom (S^2−^), whereas the remaining two electrons occupy an outer topological domain (green) located between the Li^+^ cations of the first coordination shell and the lithium cation of the second shell. The polysynaptic character of this outer domain results in a high degree of delocalization of the two electrons associated with it [49].

**FIGURE 3:**
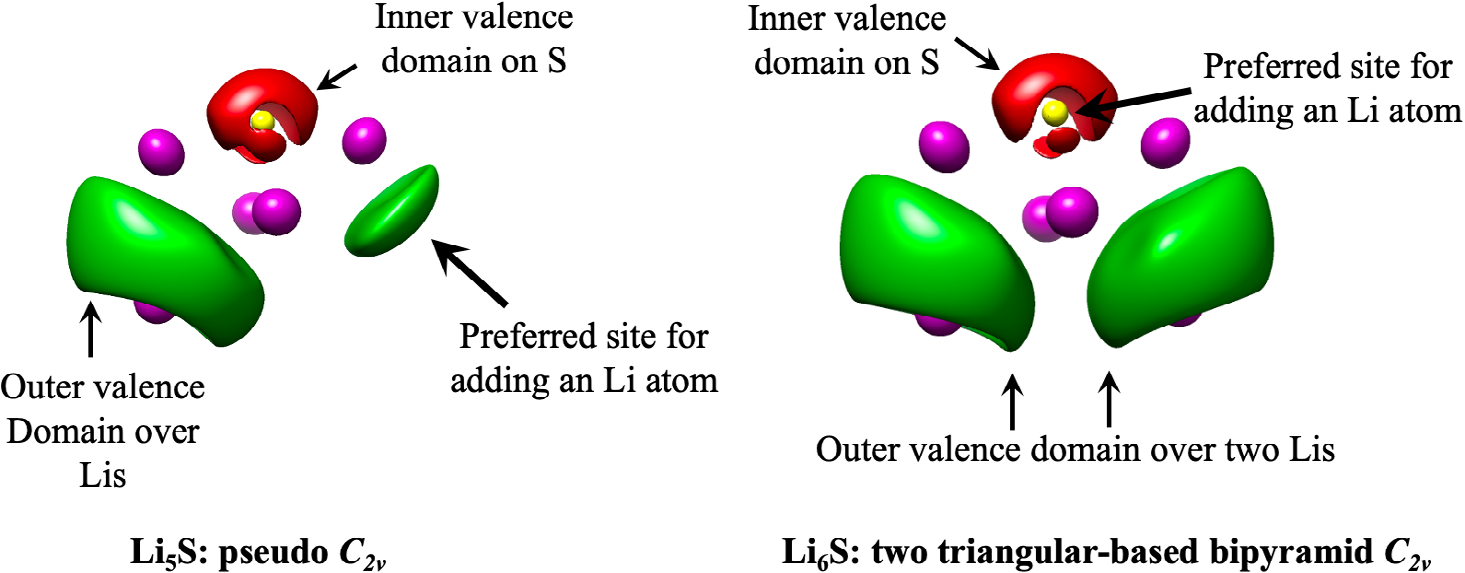
Inner and outer ELF valence domains for trigonal Li_5_S (*η*(*r*) = 0.84) (left) and Li_6_S, consisting of two edge‐sharing trigonal bipyramids (*η*(*r*) = 0.84) (right).

Since the electrons in the outer valence domain are considerably more paired (ELF = 0.988) than those in the inner valence domain (ELF = 0.861), the preferred site for the fifth incoming lithium atom is the inner valence domain. It should be noted, however, that this domain does not uniformly surround the sulfur atom. Rather, it consists of regions of high and low electron localization, giving rise to topological holes on its surface. These holes constitute the most favorable sites for accommodating an additional lithium atom (see Figure 2, right panel). Consequently, the fifth lithium atom binds to the S^2−^ center, forming a tetrahedral arrangement with the S^2−^–Li^+^–Li^+^ triangular framework (Figure 3, left panel). This structural motif is fully consistent with the global minimum structure of Li_5_S.

In the Li_5_S molecule, the sulfur atom is four‐coordinate, being surrounded by four Li^+^ ions with an average Li–S bond distance of 2.254 Å. The topological inner domain, centered on the sulfur atom and consistently containing 7.9 electrons, is consistent with the octet rule. As shown in Figure 3 (left panel), Li_5_S exhibits two distinct outer valence domains: one, almost located between the Li^+^ cations of the first coordination shell and the lithium cation of the second shell (ELF = 0.994); the other, completely delocalized, is found above the triangular facet formed by the three Li^+^ ions (ELF = 0.941). As in Li_3_S, the latter domain constitutes the preferred site for the insertion of an additional lithium atom.

In the electronic ground state of Li_6_S (C_2v_), two triangular bipyramids share a common four‐coordinate Li_4_S subunit (Figure 3, right panel). The average Li‐S bond distance is 2.261 Å, slightly longer than in Li_5_S (2.254 Å).

In Li_6_S, each of the two outer valence domains, located between the first coordination shell and a lithium cation of the second coordination shell, contains two strongly paired electrons (ELF = 0.995), whereas the inner valence domain, centered on the sulfur atom, contains eight electrons (ELF = 0.851). Consequently, as in Li_4_S, the ELF topology predicts that the preferred site for the addition of a seventh lithium atom is one of the two topological holes located on the sulfur atom and oriented perpendicular to the *σ*ᵥ molecular plane.

The evolution from Li_3_S to Li_6_S reveals that the localization or delocalization of the inner and outer valence domains dictates the preferred site for successive lithium incorporation, thereby providing a simple topological framework for understanding the growth of hyperlithiated Li–S clusters.

The geometric structure of Li_7_S can be viewed as resulting from the insertion of an additional lithium atom into Li_6_S at its preferred binding site (Figure 3, right panel). The optimized structure of Li_7_S (see Figure 1) fully supports this structural interpretation.

**FIGURE 4:**
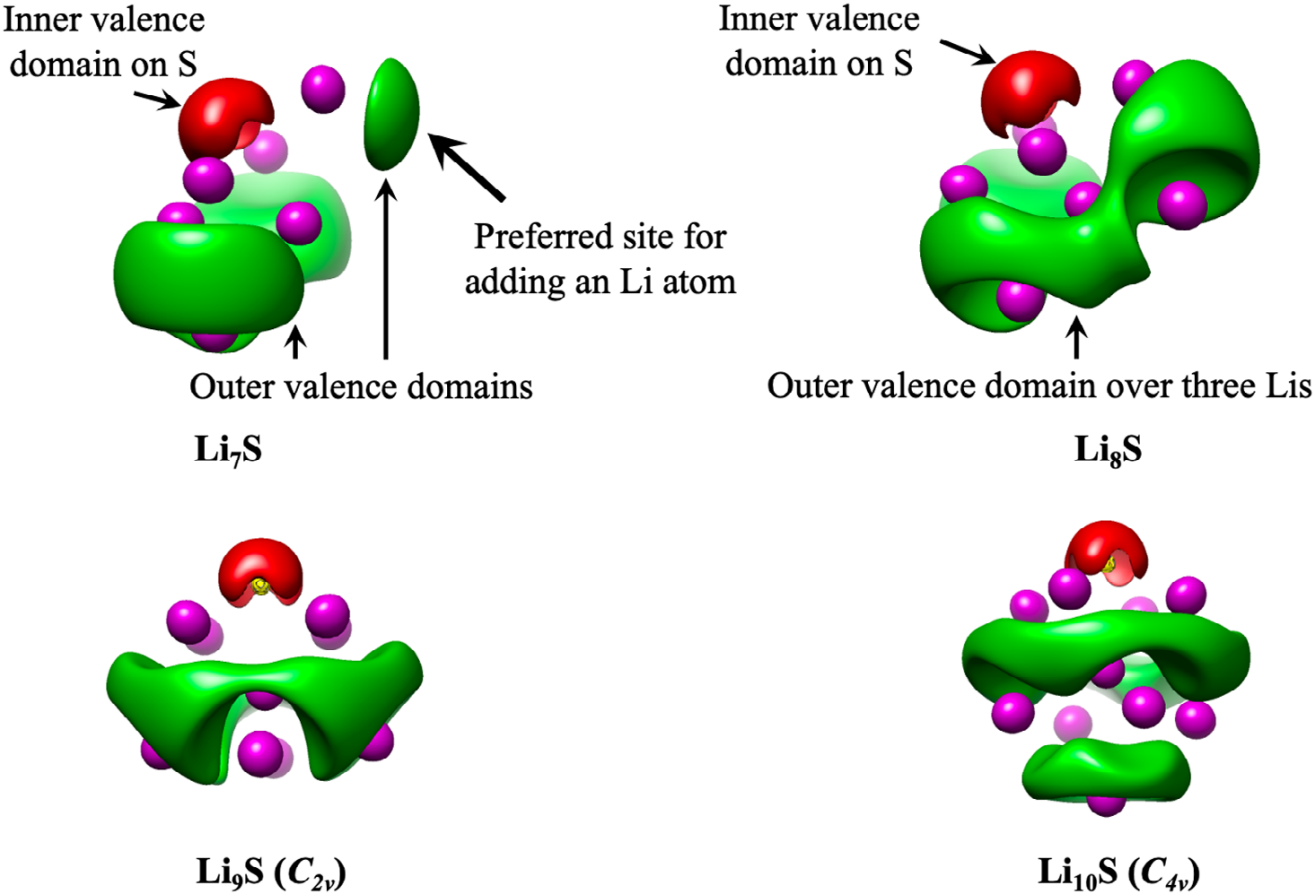
Inner and outer ELF valence domains for Li_7_S (upper left), Li_8_S (upper right), Li_9_S (lower left), and Li_10_S (lower), calculated at *η*(*r*) = 0.85.

Two noteworthy features of Li_7_S deserve emphasis. First, the sulfur atom becomes five‐coordinate, with an average Li–S bond distance of 2.331 Å, slightly longer than that in Li_6_S (2.261 Å). Although Li_7_S does not possess C_2v_ symmetry, the sulfur atom and the five lithium atoms constituting its first coordination shell adopt an arrangement that closely approximates a local C_4v_ geometry. Second, among the outer valence domains, one is completely delocalized over one of the five lithium atoms belonging to the first coordination shell. This electron‐rich domain constitutes the preferred binding site for the addition of an eighth lithium atom. As illustrated in Figure 4 (upper right), the outer valence domain separates the five‐coordinated S^2−^Li^+^_5_ core from the three lithium atoms constituting the second coordination shell.

The optimized structures of Li_9_S and Li_10_S further support this structural motif. In both species, the lithium atoms are arranged in a highly symmetrical fashion around the sulfur atom, giving rise to C_2v_ and C_4v_ symmetries for Li_9_S and Li_10_S, respectively. Notably, the sulfur atom remains five‐coordinate upon addition of the ninth and tenth lithium atoms. The average Li–S bond distance within the first coordination shell changes very little from Li_8_S to Li_9_S (2.332 vs. 2.333 Å), increasing only slightly to 2.350 Å in Li_10_S.

This behavior closely parallels the growth of lithium clusters around oxygen impurities reported previously [42]. The nearly constant average Li–S bond length from Li_8_S to Li_9_S, together with its only marginal increase in Li_10_S, indicates that the five‐coordinate sulfur environment remains remarkably robust despite the continued growth of the lithium framework up to Li_10_.

It is also noteworthy that the S^2−^ dianion remains adsorbed on the surface of the Li_*n*_^2+^ cluster, as does the O^2−^ dianion [42]. Boron, carbon, and silicon, in contrast, are endohedral over the whole size range [23, 48, 50, 51]. Consequently, the term coordination shell is not perfectly suitable in the present context. A more appropriate description is that S^2−^ (and O^2−^) behaves as a surface adsorbate, where stabilization is dominated by long‐range electrostatic interaction and the remarkable flexibility of the lithium framework.

### 3.2 Li_*n*_S_2_ (*n* = 1–10): S_2_ Dissociation Over the Lithium Clusters

**FIGURE 5:**
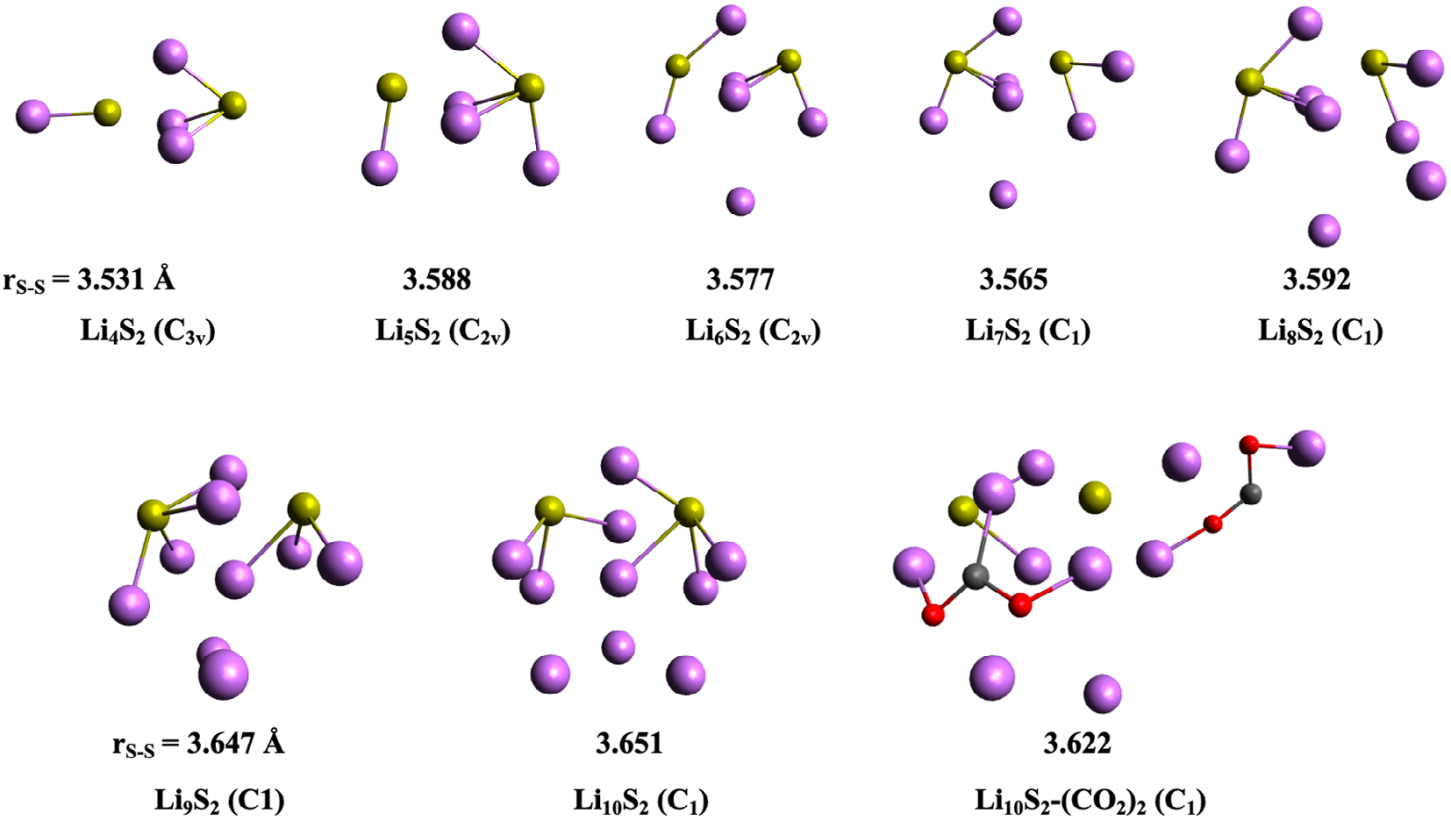
Schematic representation of the optimized geometries of Li_*n*_S_2_ (*n* = 3–10), together with the most relevant bond lengths. The purple and yellow spheres represent lithium and sulfur atoms, respectively.

The fully optimized geometric structures are depicted in Figure 5. Corresponding cartesian coordinates are reported in the Supporting Information (SM‐2).

Upon the successive addition of lithium atoms to the initial Li_4_S_2_ molecule—which obeys the octet rule—the interaction energy varies with cluster size, ranging from 25.4 kcal/mol for Li_7_S_2_ to 38.1 kcal/mol for Li_5_S_2_. The average interaction energy is 31.9 kcal/mol, which is remarkably close to the corresponding value for the Li_*n*_S series (31.1 kcal/mol). The Li_10_S_2_ cluster contains two coordination shells. As in the Li_*n*_S series, the successive formation of these shells does not follow a regular pattern but is dictated by the topological organization of the valence electrons.

**FIGURE 6:**
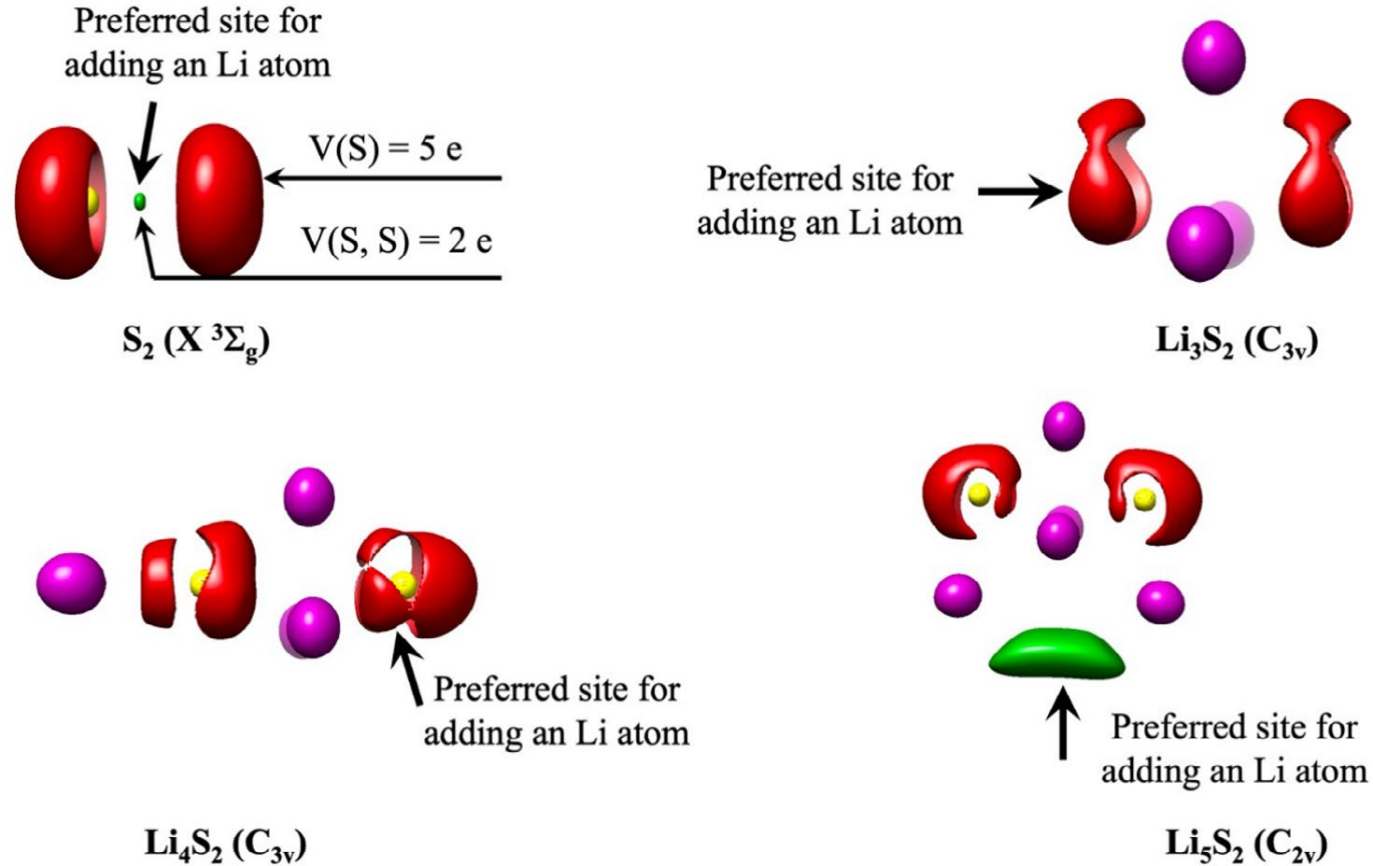
Bonding pattern in S_2_, Li_3_S_2_, Li_4_S_2_, and Li_5_S_2_. Selected results from the ELF topological analysis are also reported for each compound.

Molecular sulfur, S_2_, has a spin triplet ground state (*X* ^3^Σ_*g*_^−^) [52, 53, 54]. From the perspective of the electron localization function (ELF) theory, the bonding pattern of the S_2_ molecule consists of two monosynaptic basins, one centered on each sulfur atom, and one disynaptic basin accounting for the single S–S covalent bond. The two unpaired electrons are essentially localized on the sulfur atoms. Upon interaction with three lithium atoms, the S_2_ molecule forms a C_3v_‐symmetric structure in which the S–S bond is completely cleaved, as evidenced by the disappearance of the disynaptic S–S basin, with the three lithium atoms bridging the two sulfur atoms. Consistent with a previous study of the most stable structure of Li_4_S_2_ [13], the addition of a fourth lithium atom at a dangling position preserves the C_3v_ symmetry, yielding a compound composed of two S^2−^ anions and four Li^+^ cations. In Li_4_S_2_, the three‐coordinate sulfur atom exhibits an ELF inner valence domain‐oriented opposite to the dangling lithium atom. As shown in Figure 6, three charge‐depleted regions can be clearly identified within this inner domain, giving rise to ELF topological holes.

It is precisely this hole, characterized by a topological saddle point, that constitutes the most favorable site for the addition of an extra lithium atom. The electronic ground‐state structure of Li_5_S_2_ (C_2v_) fully supports this prediction. In Li_5_S_2_, each sulfur atom is in close contact with four lithium atoms (average Li–S distance = 2.279 Å); that is, each sulfur atom is four‐coordinate. According to the ELF analysis, the addition of a sixth lithium atom occurs at the delocalized outer valence domain, yielding a Li_6_S_2_ compound with C_2v_ symmetry. The calculated electronic ground‐state structure of Li_6_S_2_ is fully consistent with this prediction. Interestingly, the coordination number of each sulfur atom remains unchanged upon going from Li_5_S_2_ to Li_6_S_2_, indicating that the sixth lithium atom occupies the second coordination shell rather than bonding directly to either sulfur atom. The latter bears a negative charge localized within its multisynaptic basin, thereby ensuring the electrostatic balance of the compound.

Due to C_2v_ symmetry, the two sulfur atoms in the Li_6_S_2_ compound are equivalent. The topological inner domain of each sulfur atom contains two equivalent holes capable of accommodating an additional lithium atom. Starting from one of these four favorable sites, we optimized the geometry of Li_7_S_2_, which belongs to the C_1_ point group. The two sulfur centers differ in terms of coordination number: the one that accommodated the extra Li is now a five‐coordinated center with an average Li‐S distance of 2.323 Å, whereas the other S remains four‐coordinated with an average Li‐S distance of 2.268 Å.

**FIGURE 7:**
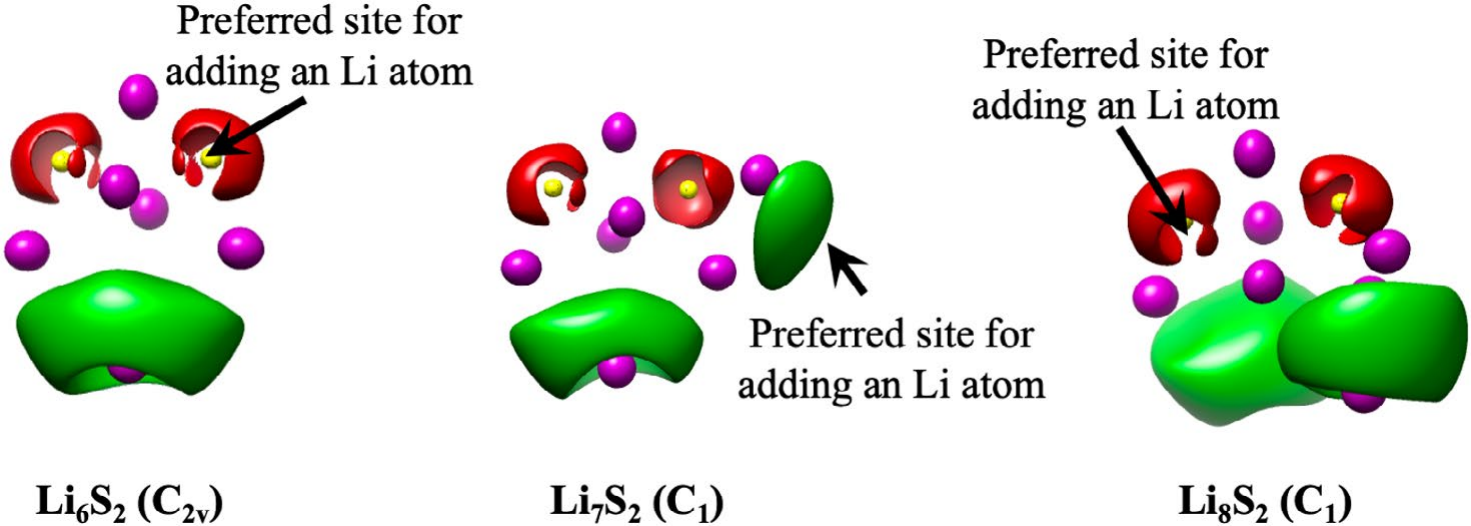
Inner (green) and outer (red) ELF domains of Li_6_S_2_, Li_7_S_2_, and Li_8_S_2_. The most favorable site for the addition of an incoming lithium atom is indicated by an arrow.

As illustrated in Figure 7, the presence of a delocalized outer domain in Li_7_S_2_ constitutes the most favorable site to accommodate the eighth lithium atom. In the optimized structure of Li_8_S_2_, the coordination numbers are preserved upon going from Li_7_S_2_ to Li_8_S_2_: one four‐coordinate (averaged Li–S = 2.270 Å) and one five‐coordinate (averaged Li–S = 2.341 Å).

It is only in the Li_9_S_2_ compound that both sulfur atoms become five‐coordinate, with an average Li–S bond distance of 2.337 Å. The addition of a tenth Li atom to the Li_9_S_2_ preserves the five‐coordinate environment around both sulfur atoms, while the average Li–S bond distance increases only slightly to 2.344 Å. In the resulting Li_10_S_2_ cluster, seven of the 10 lithium atoms belong to the first coordination.

**FIGURE 8:**
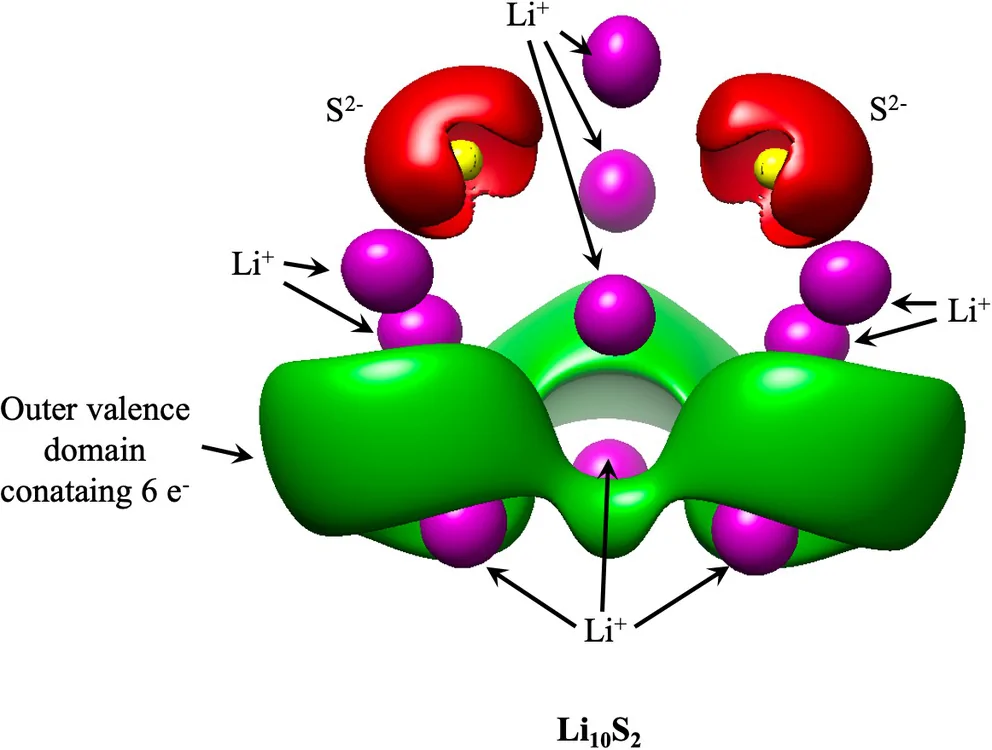
Bonding scheme of Li_10_S_2_ as deduced from the ELF topology.

To examine the distribution of the 22 valence electrons within the cluster, the ELF isosurface (*η* = 0.85) is shown in Figure 8. Eight electrons are localized in the inner topological valence domain of each sulfur atom (red), whereas the remaining six electrons occupy the outer topological valence domain (green), where they are highly delocalized over the lithium framework. These delocalized electrons play a key role in stabilizing the cluster by maintaining the two‐shell organization of the lithium framework.

Comparing Li_*n*_S and Li_*n*_S_2_ reveals that the successive development of coordination shells does not follow exactly the same pattern, highlighting the influence of the number of sulfur centers on the organization of the lithium framework. Two features are particularly noteworthy. First, in Li_*n*_S_2_, the presence of two sulfur atoms results in their separation by a three lithium‐atom barrier that persists from *n* = 3 to *n* = 10. This specific arrangement promotes the progressive increase in sulfur coordination, with both sulfur atoms becoming five‐coordinate at *n* = 9, whereas five‐coordinate sulfur is already reached at *n* = 8 in Li_*n*_S. Second, despite the larger number of atoms, Li_10_S_2_ exhibits only two distinct coordination shells, while three coordination shells are already established in Li_10_S. These differences indicate that the growth of the lithium framework is governed not simply by the number of Li atoms added, but also by the topology imposed by the sulfur centers, as revealed by the ELF topology.

Finally, we emphasize that the lithiation process of S_2_, considered here as a model system, cannot be directly compared with the reactivity of soluble lithium polysulfides (Li_2_S_8_, Li_2_S_6_, and Li_2_S_4_) in Li–S batteries. Indeed, these species can diffuse through the electrolyte and undergo a series of electrochemical reactions, leading in particular to the well‐known “shuttle effect” [55, 56, 57, 58]. A study of the lithiation of these three soluble species (Li_2_S_8_, Li_2_S_6_, and Li_2_S_4_) is currently underway.

### 3.3 Li_10_S_2_ –2CO_2_ Cluster: A Test for the Localization of Delocalized Electrons

**FIGURE 9:**
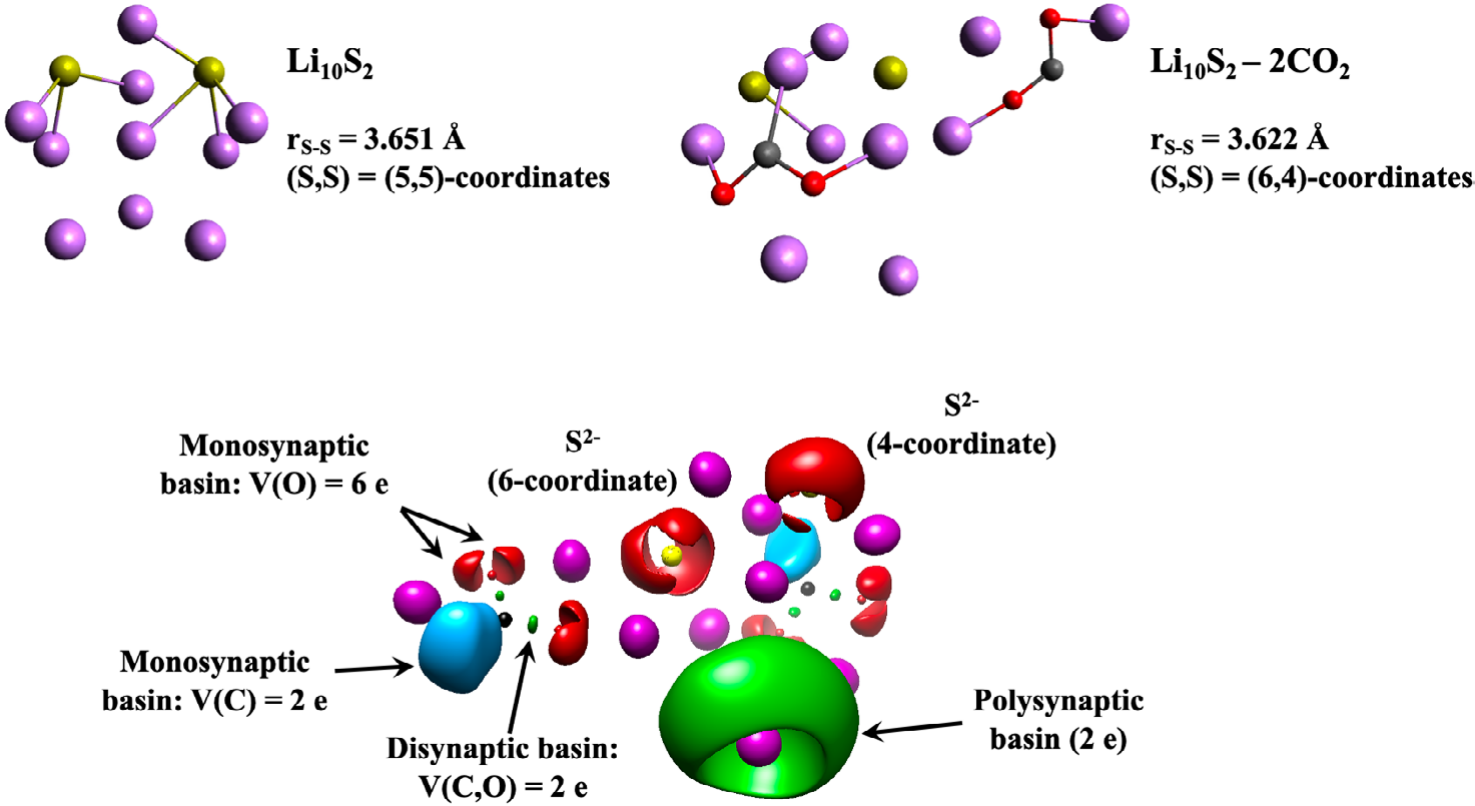
Structures (Li_10_S_2_ and Li_10_S_2_–2CO_2_) and electron distribution within the Li_10_S_2_ cluster doped by two CO_2_ molecules at ELF = 0.85.

To examine the role of the six electrons delocalized between the two lithium coordination shells, we investigated the insertion of two and three CO_2_ molecules into the Li_10_S_2_ cluster. In the case of Li_10_S_2_–3CO_2_, all geometry optimization attempts converged to dissociated structures. In contrast, the insertion of two CO_2_ molecules into Li_10_S_2_ yields a stable optimized structure in which each CO_2_ molecule is intercalated between the two lithium coordination shells, that is, within the outer valence domain (see Figure 9).

A comparison of the Li_10_S_2_ and Li_10_S_2_–2CO_2_ structures reveals three notable features. First, the S–S distance decreases slightly, by approximately 0.03 Å. Second, the two sulfur atoms become four‐ and six‐coordinate, respectively, with an average Li–S bond distance of 2.334 Å, corresponding to a slight shortening of about 0.01 Å. Third, four of the six electrons that are delocalized in Li_10_S_2_ become clearly localized on the two CO_2_ units, whereas the remaining two electrons still occupy an outer valence domain located between four lithium atoms arranged in a near‐tetrahedral geometry.

It is particularly noteworthy that the two CO_2_ species, each accommodating two electrons, are both CO_2_^2−^ dianions (carbonites) [59, 60, 61] According to the ELF analysis, these two electrons are localized in a monosynaptic valence basin, V(C), centered on the carbon atom.

The failure to stabilize a Li_10_S_2_–3CO_2_ structure is equally instructive. The electronic structure of Li_10_S_2_–2CO_2_ indicates that not all of the six delocalized electrons can be localized onto guest molecules. At least two electrons must remain delocalized within the outer valence domain, where they play a crucial role in maintaining the cohesion between the successive lithium coordination shells.

Finally, the Li_10_S_2_–2CO_2_ structure provides compelling evidence that sulfur can adopt a six‐coordinate environment, provided that the surrounding lithium framework is sufficiently favorable. This observation suggests that it would be worthwhile to investigate larger Li_*n*_S and Li_*n*_S_2_ clusters (*n* > 10) to determine the maximum coordination number that sulfur can achieve in these systems.

## 4 Conclusions

In this work, we investigated the structural and electronic evolution of the Li_*n*_S and Li_*n*_S_2_ clusters (*n* = 1–10). By following the progressive increase in sulfur coordination during cluster growth, we showed that the sulfur centers eventually become five‐coordinate in both Li_10_S and Li_10_S_2_. Throughout the entire size range investigated, however, the S^2−^ dianion is not incorporated into the lithium framework but remains adsorbed at the surface of the Li_*n*_^2+^ cluster. Accordingly, the successive Li coordination environments should not be interpreted as indicating an endohedral structure. Rather, sulfur behaves as a surface adsorbate while the lithium framework develops around it. In other words, thanks to the highly directional orientation of its valence basin (supported by the ELF analysis), sulfur directs the growth of the lithium framework.

The ELF topology revealed that the valence electrons are organized into two distinct types of topological valence domains: an inner domain localized around the sulfur atom and an outer domain associated with the lithium framework. The interplay between these inner and outer domains enables the ELF topology to identify the preferred incorporation sites for incoming lithium atoms and therefore predict the stepwise growth of the lithium framework. Taken together, these results demonstrate that the topology of the electron localization function provides a unified framework for understanding—and predicting—the stepwise growth of lithium clusters around sulfur species.

Extending this analysis to Li_*n*_S_4_ and, in particular, Li_*n*_S_8_ clusters will allow us to assess the generality of this predictive framework for increasingly complex sulfur species directly relevant to Li–S battery chemistry.

## Supporting information


**Data S1:** jcc70503‐sup‐0001‐Supinfo.docx.

## Data Availability

The data that support the findings of this study are available in the Supporting Information of this article.
