# Supplementary material for: Predicting the Stepwise Growth of Lithium Clusters Around Sulfur Species (S and S2 ) From the Topology of the Electron Localization Function
Source: J Comput Chem. 2026 Sep 9;47(24):e70503. doi: 10.1002/jcc.70503 (PMC13558790; doi:10.1002/jcc.70503)
Supplement: Supplementary file 1 [file JCC-47-0-s001.docx]

Supplementary material

**Predicting the Stepwise Growth of Lithium Clusters around Sulfur Species (S and S₂) from the Topology of the Electron Localization Function**

Mohammad Esmaïl Alikhani

Sorbonne Université, CNRS, De la Molécule aux Nano-objets: Réactivité, Interactions et Spectroscopies, MONARIS, F-75005 Paris, France

E-mail: [esmail.alikhani@sorbonne-universite.fr](mailto:esmail.alikhani@sorbonne-universite.fr) ID: <https://orcid.org/0000-0001-8412-414>7

| **SM-1: Li_n_S (n=1-10) Cartesian Coordinates** | **2** |
| --- | --- |
| a) **B3LYP GD3(BJ) / 6-311+G(2d)** b) **CCSD(T) / 6-311+G(2d)** | **2**  **3** |
| **SM-2: Li_n_S_2_ (n=1-10) Cartesian Coordinates**   a) **B3LYP GD3(BJ) / 6-311+G(2d)** | **4**  **4** |

**SM-1: Li_n_S (n=1-10) Cartesian Coordinates**

a) **B3LYP GD3(BJ) / 6-311+G(2d)**

Li2S (C2v): E = -413.352344601

S	0.000000	0.000000	0.216542

Li	0.000000	1.919233	-0.577446

Li	0.000000	-1.919233	-0.577446

Li3S (C3v): E = -420.901747281

S	0.000000	0.000000	0.379705

Li	0.000000	1.902795	-0.675031

Li	1.647869	-0.951397	-0.675031

Li	-1.647869	-0.951397	-0.675031

Li4S (C3v): E = -428.441871356	Li4S (C2v): E = -428.437863537

S	0.000000	0.000000	0.971265. S	0.000000	0.000000	0.412870

li	0.000000	1.627684	-0.489903	Li	0.000000	2.204319	0.23689

li	1.409616	-0.813842	-0.489903	Li	0.000000	-2.204319	0.236895

li	-1.409616	-0.813842	-0.489903	Li	1.420418	0.000000	-1.337881

li	0.000000	0.000000	-3.710368	Li	-1.420418	0.000000	-1.337881

Li5S: E = -435.978407475

S	0.523043	0.673384	0.000000

Li	-1.130475	2.124227	0.000000

Li	1.287811	-1.404148	0.000000

Li	-0.982300	-0.479848	1.302035

Li	-0.982300	-0.479848	-1.302035

Li	-0.982300	-3.351765	0.000000

Li6S (C2v): E = -443.520498856

S	0.000000	0.000000	1.100236

Li	0.000000	2.104359	0.453898

Li	0.000000	-2.104359	0.453898

Li	-1.259310	0.000000	-0.849352

Li	1.259310	0.000000	-0.849352

Li	0.000000	-2.307038	-2.538509

Li	0.000000	2.307038	-2.538509

Li7S: E = -451.050190737

S	0.274235	-1.028481	0.000000

Li	-1.809248	-1.815258	0.000000

Li	0.053238	-0.189005	2.102205

Li	0.053238	-0.189005	-2.102205

Li	1.350389	1.065183	0.000000

Li	-1.216679	1.009181	0.000000

Li	0.053238	2.802068	-2.040082

Li	0.053238	2.802068	2.040082

Li8S: E = -458.594817666

Li	-0.382318	-1.425931	-1.192006

Li	0.999212	1.848178	1.046507

Li	-1.534295	-2.961041	1.052815

Li	-3.108712	-0.188042	-1.005555

Li	-1.883534	2.752832	1.052565

Li	-0.998186	-0.060467	0.926250

Li	-0.552891	1.369967	-1.190772

S	1.171268	0.071065	-0.325357

Li	1.213960	-1.714511	1.045430

Li9S: E = -466.132786190

S	0.000000	0.000000	1.500610

Li	0.000000	0.000000	-0.957271

Li	1.433735	1.504409	0.511622

Li	-1.433735	-1.504409	0.511622

Li	-1.433735	1.504409	0.511622

Li	1.433735	-1.504409	0.511622

Li	0.000000	2.345451	-2.131812

Li	-2.097073	0.000000	-2.414422

Li	0.000000	-2.345451	-2.131812

Li	2.097073	0.000000	-2.414422

Li10S (C2v): E = -473.678812304

S	0.000000	0.000000	1.782639

Li	0.000000	0.000000	-0.850083

Li	0.000000	2.047901	0.782422

Li	0.000000	-2.047901	0.782422

Li	-2.047901	0.000000	0.782422

Li	2.047901	0.000000	0.782422

Li	-1.643126	1.643126	-1.973497

Li	-1.643126	-1.643126	-1.973497

Li	1.643126	-1.643126	-1.973497

Li	1.643126	1.643126	-1.973497

Li	0.000000	0.000000	-3.893028

b) **CCSD(T) / 6-311+G(2d)**

Li4S (C3v): E = -427.6732323	Li4S (C2v): E = -427.6685217

S	0.000000	0.000000	0.939974	S	0.000000	0.000000	0.457200

Li	0.000000	1.623669	-0.555077	Li	0.000000	2.208318	0.126658

Li	1.406138	-0.811834	-0.555077	Li	0.000000	-2.208318	0.126658

Li	-1.406138	-0.811834	-0.555077	Li	0.000000	-2.208318	0.126658

Li	0.000000	0.000000	-3.347965	Li	0.000000	-2.208318	0.126658

**SM-2: Li_n_S_2_ (n=1-10) Cartesian Coordinates**

a) **B3LYP GD3(BJ) / 6-311+G(2d)**

S2: E = -796.4282877

S	0.000000	0.000000	0.961352

S	0.000000	0.000000	-0.961352

LiS2 (C2v): E = -804.0246642

S	0.000000	1.023697	-0.169752

Li	0.000000	0.000000	1.810692

S	0.000000	-1.023697	-0.169752

Li2S2 (C2v): E = -811.6264497

S	0.134203	0.081312	1.112845

Li	-1.565699	0.969273	0.000000

S	0.134203	0.081312	-1.112845

Li	0.134203	-1.836601	0.000000

Li3S2 (C3v): E = -819.2187925

s	1.552771	0.000144	-0.000069

li	-0.000321	1.500305	-0.675728

s	-1.552545	0.000176	-0.000056

li	-0.000513	-1.336804	-0.960697

li	-0.000370	-0.165210	1.637091

Li4S2 (C3v): E = -826.811484

s	-1.519255	-0.001269	0.000067

li	0.333670	-0.538973	1.362486

li	-3.625929	0.004084	-0.000239

s	2.011600	0.000590	-0.000040

li	0.333806	-0.911382	-1.147545

li	0.332615	1.449891	-0.214849

Li5S2 (C2v): E = -834.3635585

li	0.000000	0.000000	1.712125

s	0.000000	1.793895	0.275213

s	0.000000	-1.793895	0.275213

li	0.000000	2.155772	-1.871670

li	0.000000	-2.155772	-1.871670

li	-1.280130	0.000000	-0.452194

li	1.280130	0.000000	-0.452194

Li6S2 (C2v): E = -841.908115

S	1.788702	-0.541020	0.000004

Li	2.481178	1.495202	0.000225

Li	0.000899	-1.936944	-0.000999

S	-1.788202	-0.541880	-0.000011

Li	-2.482090	1.493766	0.000280

Li	-0.000478	0.324155	1.237939

Li	-0.000477	0.325630	-1.237122

Li	-0.001698	4.073657	-0.000283

Li7S2: E = -849.4399158

S	-1.635868	-0.381124	-0.296804

Li	-2.158669	1.812182	-0.100028

Li	0.062305	-1.923501	-0.162639

S	1.893992	-0.686781	0.100977

Li	2.835679	1.260907	0.160994

Li	0.379448	0.468041	-1.263469

Li	0.123544	0.360823	1.210303

Li	0.668020	4.061719	0.000000

Li	-3.286989	-0.344677	1.199252

Li8S2: E = -856.9814149

S	-1.377733	-0.951277	-0.551688

Li	-2.134212	1.177842	-0.730978

Li	-2.706699	-0.548304	1.202964

Li	0.510043	-1.978004	0.224732

S	2.043011	-0.374883	0.380570

Li	2.650014	1.648664	-0.070298

Li	-0.026475	0.555739	0.984627

Li	0.594645	0.048943	-1.416388

Li	0.196994	3.883070	-1.298646

Li	-2.632464	2.284905	2.016614

Li9S2: E = -864.5191768

S	-1.678772	-0.460398	-0.787666

Li	-1.830137	-1.224123	1.379459

Li	-2.453827	1.380090	0.260284

Li	0.129897	-1.822174	-0.582503

S	1.909413	-0.460218	-0.133254

Li	2.558691	1.646373	-0.246210

Li	0.219400	0.589134	-1.636491

Li	-0.025423	0.603880	0.779459

Li	-1.232564	0.814641	3.306734

Li	0.064758	4.149589	-0.327662

Li	1.339116	-1.227460	1.978503

Li10S2: E = -872.0648382

S	1.827051	-0.943794	0.032466

Li	0.003410	-1.975795	-0.777615

S	-1.824168	-0.948661	0.031431

Li	-2.518671	0.461689	-1.558701

Li	2.518979	0.465496	-1.559098

Li	-2.266818	0.995010	1.137922

Li	2.264119	1.001296	1.138361

Li	0.000425	-0.741810	1.543328

Li	-0.000366	0.821424	-0.394671

Li	-1.653835	3.192969	-0.886376

Li	1.641094	3.195325	-0.892084

Li	-0.003714	2.677493	1.908151

Li10S2-2CO2: E = -1249.60282263

S	0.579447	-0.394692	-0.523559

Li	2.873985	-0.296834	-0.857920

Li	-0.185759	-2.526160	-0.017132

S	-2.323366	-2.313624	0.481485

Li	-1.740349	-0.817257	-1.257775

Li	1.774467	0.600240	1.289308

Li	-3.947611	-0.794796	0.238031

Li	-1.174211	-0.463430	1.268904

Li	-0.448813	1.684281	-0.675571

Li	-0.607607	2.567365	3.251708

Li	6.425397	0.063069	-0.250341

Li	-3.153264	2.567486	0.630180

C	-2.800844	0.982113	-0.846637

O	-3.888974	1.011100	-0.113937

O	-2.195838	2.140735	-0.915322

C	4.672113	0.336861	0.429150

O	4.754018	-0.207126	-0.788335

O	3.484093	0.513707	0.857584

CO2: E = -188.652282988

C	0.000000	0.000000	0.000000

O	0.000000	0.000000	1.160342

O	0.000000	0.000000	-1.160342
